# Performance of Single-Shot Echo-Planar Imaging in Diffusion Tensor Imaging in Rat Sciatic Nerve Compared With Readout-Segmented Echo-Planar Imaging

**DOI:** 10.3389/fnins.2022.844408

**Published:** 2022-05-12

**Authors:** Yueyao Chen, Zhongxian Pan, Fanqi Meng, Zhujing Li, Yuanming Hu, Xuewen Yu, Jinyun Gao, Yihao Guo, Hanqing Lyu, Xiaofeng Lin

**Affiliations:** ^1^Department of Radiology, Shenzhen Traditional Chinese Medicine Hospital, The Fourth Clinical Medical College of Guangzhou University of Chinese Medicine, Shenzhen, China; ^2^Department of Pathology, Shenzhen Traditional Chinese Medicine Hospital, The Fourth Clinical Medical College of Guangzhou University of Chinese Medicine, Shenzhen, China; ^3^Magnetic Resonance Collaboration, Siemens Healthcare, Guangzhou, China; ^4^Department of Nuclear Medicine, The Seventh Affiliated Hospital, Sun Yat-sen University, Shenzhen, China

**Keywords:** magnetic resonance imaging, diffusion tensor imaging, single-shot echo-planar imaging, readout-segmented echo-planar imaging, peripheral nerve

## Abstract

**Objectives:**

To compare the performances of single-shot echo-planar imaging (SS–EPI) and readout-segmented echo-planar imaging (RS–EPI) for diffusion tensor imaging (DTI) of the rat sciatic nerve.

**Methods:**

Eight healthy adult male Sprague-Dawley rats were anesthetized and scanned with a 3T MRI scanner using SS–EPI and RS–EPI DTI sequences. The image quality in terms of the morphology of the nerve, distortions of the nearby femur, muscles, and homogeneity of neuromuscular were evaluated and scored. The correlations between the DTI parameters including fractional anisotropy (FA), axial diffusivity (AD), radial diffusivity (RD), apparent diffusion coefficient (ADC), and histopathological parameters were calculated by using the Pearson correlation coefficient and compared by the modified Fisher *Z*-transform, respectively.

**Results:**

The quality scores were higher for the images from the SS–EPI sequence compared with the RS–EPI sequence for characteristics such as sharpness of the sciatic nerve margin (*P* = 0.008), artifacts of the sciatic nerve (*P* = 0.008), and homogeneity of the neuromuscular region (*P* = 0.007), as well as the contrast-to-noise ratio (CNR) of DW images (*P* < 0.001). The correlation coefficients were higher for the FA and RD values from the SS–EPI sequence compared with those from the RS–EPI sequence. Furthermore, the correlation coefficients between FA and myelin thickness (*P* = 0.027), FA and diameter of the myelinated fiber (*P* = 0.036), as well as RD and myelin thickness (*P* = 0.05) were statistically higher for the SS–EPI sequence compared with those for the RS–EPI sequence.

**Conclusion:**

Diffusion tensor imaging analysis of the rat sciatic nerve showed that the image quality from the SS–EPI sequence was significantly higher compared with that from the RS–EPI sequence. Furthermore, the FA and RD derived from the SS–EPI sequence are promising and sensitive biomarkers to detect the histopathological changes in the rat sciatic nerve.

## Introduction

Diffusion tensor imaging (DTI) is a functional non-invasive MRI sequence that is widely used to evaluate cerebral white matter fiber tracts in the brain. DTI is also routinely used to assess the integrity of the peripheral nerves, especially in experimental animal studies of nerve injury. Several preclinical and animal studies have shown that DTI parameters such as fractional anisotropy (FA) and radial diffusivity (RD) are associated with axon density and myelin abnormalities (Chen et al., 2017; Farinas et al., 2020; Zheng et al., 2021). Therefore, DTI parameters such as FA and RD are highly sensitive biomarkers for the early detection of peripheral nerve dysfunction and monitoring the progression of peripheral nerve degeneration and regeneration (Chen et al., 2017; Farinas et al., 2020; Zheng et al., 2021).

Deep and prolonged anesthesia significantly increases the risk of animal death during *in-vivo* MRI studies. Furthermore, the risk of death from anesthesia is higher while performing continuous MRI to monitor peripheral nerve repair because the same animal is anesthetized and revived several times during the examination. Hence, to minimize the risk of death from anesthesia, DTI needs to be carried out in a shorter duration while ensuring sufficient quality. Furthermore, higher resolution is required for optimal analysis of the sciatic nerves in small experimental animals. Therefore, scan time, nerve display, and parameter accuracy are critically important while using DTI for peripheral nerve MRI studies in experimental animals.

Single-shot echo-planar imaging (SS–EPI) and readout-segmented echo-planar imaging (RS–EPI) are the two main types of DTI sequences. The conventional SS–EPI is more prone to susceptibility artifacts such as geometric distortions, image blurring, and ghosting artifacts (Drake-Pérez et al., 2018). Therefore, readout-segmented echo-planar imaging (RS–EPI) was developed to reduce distortion artifacts and increase image resolution (Porter and Heidemann, 2009). Better performance of the RS–EPI sequence in terms of image quality and lesion detection has improved the diagnostic performance of DTI in the brain (Kida et al., 2016), skull base and orbit (Yeom et al., 2013; Chen et al., 2020), breast (Bogner et al., 2012), kidney (Friedli et al., 2017), pelvis (Thian et al., 2014), and the sacroiliac joint (Zhang et al., 2021). However, RS-EPI significantly increases the scan time and is sensitive to movement artifacts. These two characteristics are the main obstacles in animal MRI studies.

In our preliminary study, the image quality from the SS–EPI sequence was significantly higher compared with the RS–EPI sequence for the diffusion tensor imaging of sciatic nerve repair in rats. In the liver and pediatric brains, SS–EPI sequences showed better image quality with reduced susceptibility and motion artifacts compared with the RS–EPI sequence (Yeom et al., 2013; Xie et al., 2021). However, the performances of SS–EPI DTI and RS–EPI DTI have not been compared with assessing peripheral nerves in small animals. Therefore, in this study, we compared the image quality of the sciatic nerve between the SS–EPI and RS–EPI sequences. We also analyzed the correlation between DTI parameters from the SS–EPI and RS–EPI sequences and the histological parameters of the rat sciatic nerve to identify morphological and histological changes in the rat sciatic nerve.

## Materials and Methods

### Subjects

All interventions and animal care procedures were performed in accordance with the Guidelines and Policies and were approved by the Institutional Animal Use and Care Committee. All animals were obtained from the Animal Experiment Center of Guangdong province. The animals were housed in a standard animal facility with 12-h on/off light conditions and allowed free access to standard food and water. Eight male adult healthy Sprague-Dawley rats weighing 250 ± 20 g were used in this study.

### Magnetic Resonance Imaging

In total, eight healthy adult male Sprague-Dawley rats were anesthetized to deep sleep (7% chloralhydrate, 5 ml/kg, intraperitoneal injection) and scanned at 3T (MAGNETOM Prisma, Siemens Healthcare, Erlangen, Germany). After anesthesia, each rat was placed prone in a rat coil (6-cm diameter, 8 channel, Suzhou Medcoil Healthcare Co., Ltd.) with their limbs fixed with medical adhesive tape to further prevent movement. Both hind limbs were positioned symmetrically.

**FIGURE 1:**
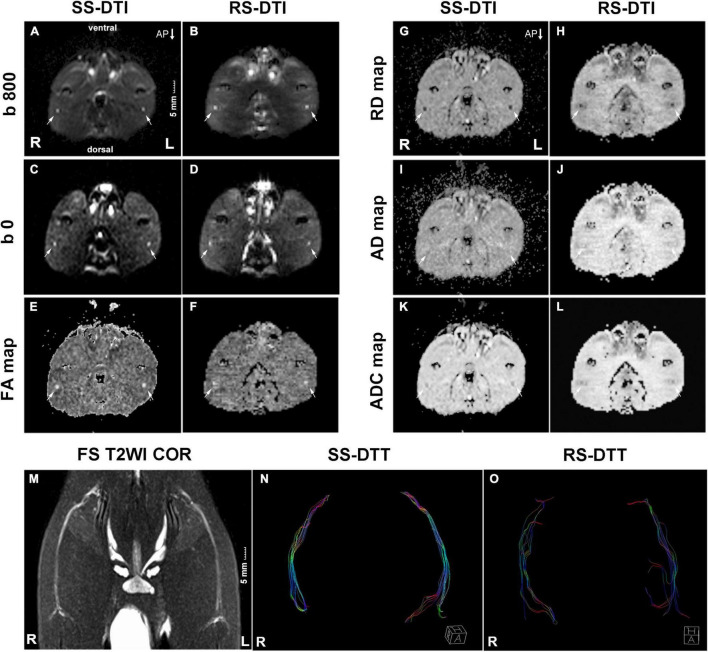
MRI images. **(A–L)** The transverse DTI images of the rat sciatic nerve in SS–DTI and RS–DTI showing different image qualities. All DTI images derived from SS–DTI had better quality than that from RS–DTI. Artifacts of the ventral and dorsal side for SS–DTI were slightly more obvious. **(M)** T2WI coronal view displayed the morphology of bilateral sciatic nerves. **(N,O)** DTT: SS–EPI DTI can generate more fibers compared with RS–EPI DTI. They displayed a more condensed bundle and realistic architecture. Conversely, fibers generated by RS–EPI DTI were sparse and discrete, some of which were deviated and in random order. R, right hind limb; L, left hind limb; AP, phase encoding direction. Scale: 5 mm.

Two Axial DTI sequences (SS–EPI and RS–EPI) were performed according to the parameters as follows (see Table 1 for detailed parameters): SS–EPI DWI (TE/TR 72/3,500 ms, slice thickness 1.5 mm, the field of view 70 × 70, matrix size 100 × 100, voxel size 0.7 mm × 0.7 mm, *b*-values 0 and 800 s/mm^2^, readout segments 1); RS–EPI DWI (TE/TR 58/4,000 ms, slice thickness 1.5 mm, the field-of-view 70 × 70, matrix size 100 × 100, voxel size 0.7 mm × 0.7 mm, *b*-values 0 and 800 s/mm^2^, readout segment 7). The axial plane was perpendicular to the long axis of the sciatic nerve. A coronal fat-suppressed T2-weighted image was obtained to display the morphology of bilateral sciatic nerves and to ensure the correct position of the region of interest (ROI) for DTI parameters measurement in the sciatic nerve (see Figure 1).

**TABLE 1:** Sequence parameters for single-shot and readout-segmented echo-planar imaging.

Sequence parameter	Single-shot echo-planar imaging (SS-EPI)	Readout-segmented echo-planar imaging (RS-EPI)
Diffusion mode	MDDW	MDDW
Diffusion directions	20	20
Diffusion schema	Monopolar	Monopolar
*b*-value (s/mm^2^)	0, 800	0, 800
Fat suppression	fat saturation	fat saturation
Repetition time (ms)	3,500	4,000
Echo time (ms)	72	58
Field of view (mm^2^)	70 × 70	70 × 70
Voxel size (mm^2^)	0.7 × 0.7	0.7 × 0.7
Slice thickness (mm)	1.5	1.5
Matrix	100 × 100	100 × 100
No. of sections	20	20
Section thickness (mm)	1.5	1.5
Intersection gap (%)	0	0
Phase-encoding direction	Anteroposterior	Anteroposterior
Echo spacing (ms)	1.13	0.54
No. of readout segments	1	7
Acquisiton time (min:s)	4:06	14:12

### Image Analysis

Image analysis was performed by two independent readers (Reader 1, a radiologist with 10 years of experience in DTI of the nerve; Reader 2, with 3 years of experience in DTI of the nerve). Both readers were blinded to the histopathologic results. Data of the two DTI sequences were transferred to the workstation (*Syngo Via* 2, Siemens) and post-processed for image quality evaluation and DTI parameters measurement. DTI images including *b* = 0, 800 images, FA, AD, RD, and ADC maps were generated simultaneously in Neuro 3D modules. The image quality was assessed qualitatively on DWI *b* = 800 image based on the following factors: sharpness of nerve margin, artifacts of nerve, artifacts of the femur, artifacts of ventral muscles, artifacts of dorsal muscles, homogeneity of neuromuscular. All images were rated according to a scale: 1, unacceptable image quality severely deteriorated by artifacts; 2, acceptable image quality with mild artifacts; 3, artifact-free image without distortions and with great anatomic detail (Thian et al., 2014). Contrast-to-noise ratio (CNR) was defined as the difference between the mean signal intensity of the nerve (S nerve) and that of the muscle surrounding on DW images (S muscle) divided by the SD on the lesion ROI (σ nerve) and normal tissue ROI (σ muscle) of the subtraction dataset as follows (Bogner et al., 2012)


CNR=Snerve-Smuscleσnerve2-σmuscle2

The diffusion tensor parameters (FA, AD, RD, and ADC) were measured and calculated on the corresponding map generated on a workstation (*Syngo Via* 2, Siemens). ROIs of approximately 4 mm^2^ were manually drawn on the adjacent 3 slices of the sciatic nerve. The three measurements were averaged for data analysis. Special attention was paid to positioning the ROIs as precisely as possible to minimize the partial volume effect. Transverse DTI images were linked with coronal T2-weighted images to ensure the correct and consistent position of ROIs in the sciatic nerve. Tractography was obtained on the same workstation. A multiple ROI method was used to reconstruct diffusion tensor tractography (DTT). The threshold of FA was set to be 0.15, the maximum fiber angle was 35° and the minimum fiber length was 15 mm (Chen et al., 2017).

### Histopathologic Assessment

Animals were executed after MRI by transcardial perfusion with PBS followed by 4% paraformaldehyde in 0.1 M PBS (pH 7.4). The middle stumps of the sciatic nerves were harvested and postfixed in 4% glutaraldehyde. As shown in Figures 5A,B, transverse semi-thin sections (1 μm thickness) were prepared and stained with toluidine blue to detect nerve myelin. For the quantification of toluidine blue staining, sections of the middle stumps were analyzed morphometrically. In brief, an objective magnification of ×1,000 was used to take digital images of the entire cross-sectional area of the nerve (60 × 40 μm, 13.7 pixels/μm) on a microscope (Olympus BX60, Japan) for detailed histological quantification. Of these images, five randomly selected measured images (per image area, 240 μm^2^; total area, 1,200 μm^2^ of different regions per nerve segment and animal were analyzed, as described previously. ImageJ software^1^ was used to perform analysis to determine the percentage of axon area (POAA), percentage of myelin area (POMA), thickness of myelin (TOM), and diameter of myelinated fibers (DOMF). The final value used for statistical analysis represents the mean of five measuring images per nerve segment and animal.

### Statistical Analysis

The image quality scores between SS–EPI and RS–EPI were compared using the Wilcoxon rank-sum test on several aspects (sharpness of nerve margin, artifacts of nerve, artifacts of femur, artifacts of ventral muscles, artifacts of dorsal muscles, and homogeneity of neuromuscular). The inter-observer variability between the two radiologists for the scores was evaluated using a linear-weighted inter-rater agreement (Kappa) test with a calculation of 95% CI. The values of Kappa over 0.75, from 0.40 to 0.75, and below 0.4 were regarded as excellent, fair to good, and poor, respectively. The Shapiro–Wilk test showed that all DTI parameters were normally distributed (*P* > 0.05 for all parameters). Differences in these parameters (FA, AD, RD, and ADC) between SS–EPI and RS–EPI were compared using the paired *t*-test. The degree of association between DTI parameters and histopathological parameters was calculated by using the Pearson correlation coefficient. These coefficients of SS–EPI and RS–EPI were then compared by using the modified Fisher *Z*-transform (Meng et al., 2014; Friedli et al., 2017). A two-sided *P*-value of 0.05 or less indicated a significant result. Statistical analysis was performed by using SPSS (version 23, IBM SPSS, Chicago) and MedCalc (version 19.1.2, MedCalc Software bv, Ostend, Belgium). Plots were created by using GraphPad Prism (Version 7.0) and RStudio (Version 1.4.1717).

## Results

### Morphology of Rat Sciatic Nerve

The bilateral sciatic nerve was best displayed on the coronal view of T2WI STIR. They split above the knee joint into tibial and peroneal nerves, and 2–3 sub-branches can be shown downward (Figure 1M). In our study, the thickness of the normal rat sciatic nerve trunk ranged from 1 mm to 2 mm.

### Comparison of Image Quality

**FIGURE 2:**
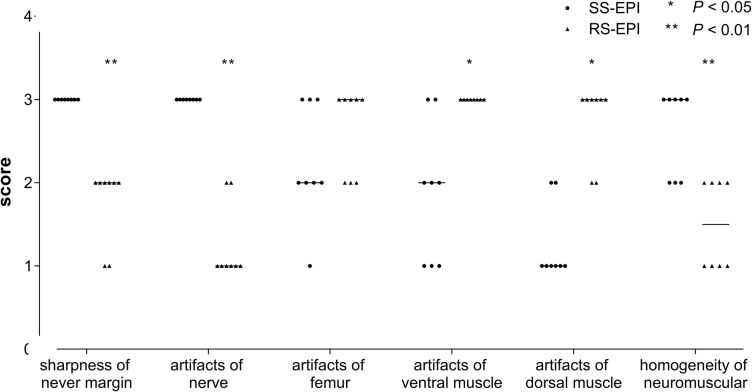
Score of image quality in both SS–EPI and RS–EPI. The chart showed the image quality scores rated by reader 1 (the more senior radiologist). The image quality scores of SS–EPI were significantly higher than those of RS–EPI.

**FIGURE 3:**
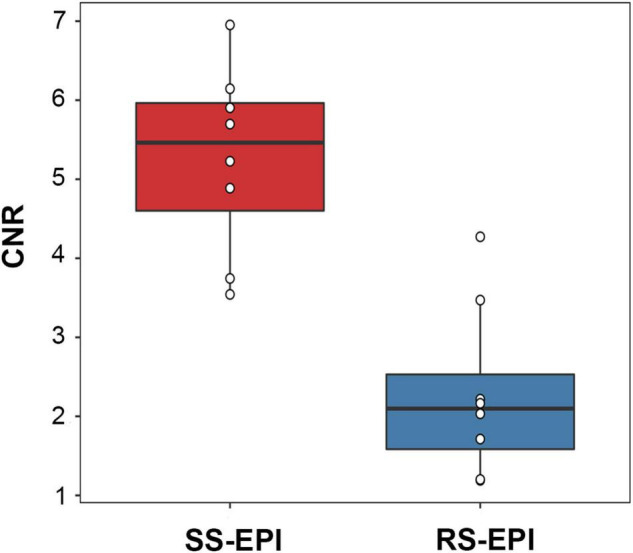
Box plot comparing the CNR (*b* = 800 s/mm^2^) of SS–EPI and RS–EPI. The CNR of SS–EPI was significantly higher than that of RS–EPI (*P* < 0.001).

The image quality scores of the two DTI images for the sharpness of nerve margin, artifacts of nerve, artifacts of the femur, artifacts of ventral muscles, artifacts of dorsal muscles, and homogeneity of neuromuscular are shown in Table 2 and Figures 1A–L, 2. The interobserver agreement between the two independent radiologists was excellent (Kappa = 0.837–0.937). Figure 2 showed the image quality scores rated by reader 1 (the more senior radiologist). The image quality scores of SS–EPI were significantly higher than those of RS–EPI in terms of blurring of nerve margin (*P* = 0.008), artifacts of the nerve (*P* = 0.008), and homogeneity of neuromuscular (*P* = 0.007). RS–EPI scored higher than SS–EPI with regard to artifacts of ventral muscles (*P* = 0.024) and artifacts of dorsal muscles (*P* = 0.010). As shown in Figure 3, the CNR of SS–EPI was significantly higher than that of RS–EPI (5.26 ± 1.17 vs. 2.28 ± 1.07, *P* < 0.001).

**TABLE 2:** Comparisons of image quality scores and inter-observer agreements between SS–EPI and RS–EPI.

Image quality evaluation	Reader 1		Reader 2		Kappa (95%CI)
	SS-EPI/RS-EPI Median (range)	*P*-value	SS-EPI/RS-EPI Median (range)	*P*-value	
Sharpness of the nerve margin	3 (3–3)/2 (1–2)	0.008*	3 (2–3)/2 (1–2)	0.008*	0.837 (0.626–1.000)
Artifacts of the nerve	3 (3–3)/1 (1–2)	0.008*	3 (3–3)/1 (1–2)	0.007*	0.937 (0.817–1.000)
Artifacts of the femur	2 (1–3)/3 (2–3)	0.257	2 (1–3)/2.5 (2–3)	0.414	0.899 (0.703–1.000)
Artifacts of the ventral muscles	2 (1–3)/3 (3–3)	0.024*	2 (1–3)/3 (2–3)	0.023*	0.921 (0.766–1.000)
Artifacts of the dorsal muscles	1 (1–2)/3 (2–3)	0.010*	1 (1–2)/2.5 (2–3)	0.015*	0.862 (0.684–1.000)
Homogeneity of the neuromuscular region	3 (2–3)/1.5 (1–2)	0.007*	2.5 (2–3)/1.5 (1–2)	0.011*	0.920 (0.766–1.000)

*Data are listed for both readers as reader1/reader 2.*

*Two independent readers rated the parameters on a scale from 1 (low) to 3 (high).*

*SS-EPI, single-shot echo-planar imaging; RS-EPI, readout-segmented echo-planar imaging; CI, confidence interval.*

*P ≤ 0.05.

### Comparison of Diffusion Tensor Imaging Parameters and Diffusion Tensor Tractography

**FIGURE 4:**
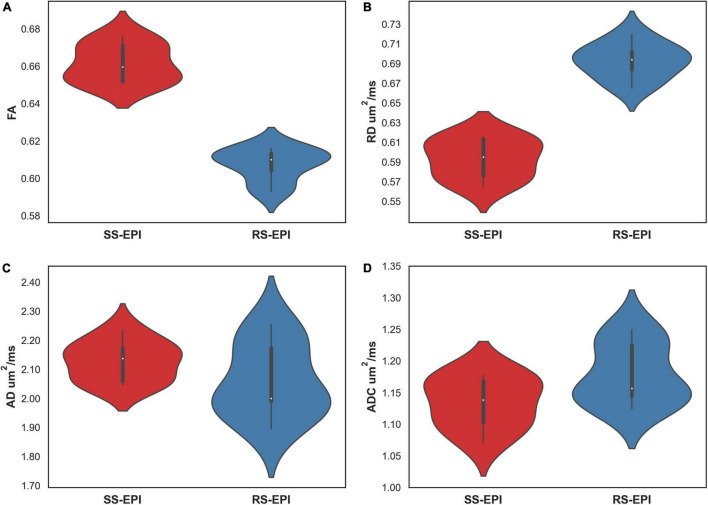
Violin plot comparing the DTI parameters of SS–EPI and RS–EPI. **(A)** The FA of SS–EPI was significantly higher than that of RS–EPI. **(B)** The RD of SS-EPI was significantly lower than that of RS–EPI. (*P* < 0.001). **(C,D)** There was no significant difference between SS–EPI and RS–EPI for ADC and AD (*P* > 0.05).

Figure 4 shows the comparison of DTI parameters between SS–EPI and RS–EPI. The FA of SS–EPI was significantly higher than that of RS–EPI (0.661 ± 0.010 vs. 0.607 ± 0.009, *P* < 0.001), and the RD of SS–EPI was significantly lower than that of RS–EPI (0.593 μm^2^/ms ± 0.020 vs. 0.693 μm^2^/ms ± 0.018, *P* < 0.001). There were not significantly different between SS–EPI and RS–EPI for ADC (1.134 μm^2^/ms ± 0.040 vs. 1.179 μm^2^/ms ± 0.048, *P* = 0.061) and AD (2.128 μm^2^/ms ± 0.068 vs. 2.060 μm^2^/ms ± 0.127, *P* = 0.280).

DTT: SS–EPI DTI can generate more fibers compared with RS–EPI DTI. They displayed a more condensed bundle and realistic architecture. Conversely, fibers generated by RS–EPI DTI were sparse and discrete, some of which were deviated and in random order.

### Correlation Between Diffusion Tensor Imaging Parameters and Histopathological Parameters

Table 3 shows the correlation coefficients between DTI parameters and histopathological parameters for both SS–EPI and RS–EPI. For both sequences, FA was correlated with all of the histopathological parameters (*r* = 0.913–0.963 for SS–EPI, and *r* = 0.741–0.897 for RS–EPI, *P* ≤ 0.001∼0.036), except the diameter of myelinated fibers for RS–EPI (*r* = 0.623, *P* = 0.099); and RD was correlated with all of the histopathological parameters (*r* = −0.886 to −0.948 for SS–EPI, and *r* = −0.709 to −0.812 for RS–EP, *P* ≤ 0.001∼0.049), except the percentage of axon area for RS-EPI (*r* = −0.691, *P* = 0.058). ADC derived from the SS–EPI sequence was related to all of the histopathological parameters (*r* = −0.738 to −0.769, *P* ≤ 0.026∼0.037), except the diameter of myelinated fibers (*r* = −0.686, *P* = 0.060); While ADC from the RS–EPI sequence was not correlated with any of the histopathological parameters (all *P* > 0.05). No statistical correlation was found between AD for either sequence and any of the histopathological parameters (all *P* > 0.05).

**TABLE 3:** Correlation coefficients between DTI parameters and histopathological parameters.

	FA		*P*-value	RD		*P*-value	AD		*P*-value	ADC		*P*-value
	SS-EPI	RS-EPI		SS-EPI	RS-EPI		SS-EPI	RS-EPI		SS-EPI	RS-EPI	
POAA	0.913 (*P* = 0.002*)	0.897 (0.003*)	0.853	−0.898 (0.002*)	−0.691 (0.058)	0.158	−0.298 (0.474)	−0.226 (0.591)	0.916	−0.769 (0.026*)	−0.232 (0.581)	0.214
POMA	0.952 (<0.001*)	0.769 (0.026*)	0.077	−0.948 (<0.001*)	−0.812 (0.014*)	0.160	−0.415 (0.307)	−0.082 (0.847)	0.631	−0.753 (0.031*)	−0.193 (0.647)	0.208
TOM	0.963 (<0.001*)	0.741 (0.036*)	0.027*	−0.944 (<0.001*)	−0.709 (0.049*)	0.050*	−0.377 (0.357)	−0.089 (0.834)	0.681	−0.738 (0.037*)	−0.144 (0.734)	0.196
DOMF	0.923 (0.001*)	0.623 (0.099)	0.036*	−0.886 (0.003*)	−0.716 (0.046*)	0.245	−0.400 (0.326)	0.005 (0.990)	0.566	−0.686 (0.060)	−0.073 (0.863)	0.209

*The independent P-value is the result of comparison of correlated correlations. The P-value in parentheses is the result of correlation.*

*DTI, Diffusion tensor imaging; FA, fractional anisotropy; RD, radial diffusivity; AD, axial diffusivity; ADC, apparent diffusion coefficient; SS-EPI, single-shot echo-planar imaging; RS-EPI, readout-segmented echo-planar imaging; POAA, percentage of axon area; POMA, percentage of myelin area; TOM, thickness of myelin; DOMF, diameter of myelinated fiber.*

*P ≤ 0.05.

**FIGURE 5:**
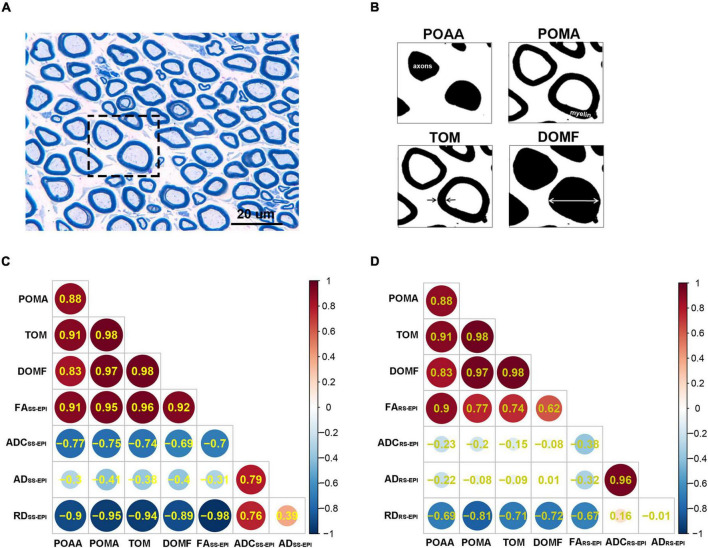
**(A)** Toluidine blue myelin staining of the sciatic nerves (×1,000). Scale: 20 μm. **(B)** Toluidine blue myelin staining were analyzed by ImageJ software to determine the quantitative pathological parameters including POAA: percentage of axon area, POMA: percentage of myelin area, TOM: thickness of myelin, and DOMF: diameter of myelinated fibers. **(C,D)** Heatmaps of Correlation between DTI parameters and histopathological parameters. The number in each circle represents the correlation coefficients (*r*-value) between the corresponding parameters. The overall correlation coefficients between SS–EPI DTI parameters and histopathological parameters **(C)** were superior to the correlation coefficients between RS–EPI DTI parameters and histopathological parameters **(D)**.

The difference between two correlation coefficients obtained from SS–EPI and RS–EPI was assessed and the results are shown in Table 3 and Figure 5. The overall correlation coefficients of FA and RD obtained with SS–EPI were numerically higher than that with RS–EPI, and the coefficients between FA and myelin thickness (*P* = 0.027), FA and myelinated fiber diameter (*P* = 0.036), and RD and myelin thickness (*P* = 0.05) for SS–EPI were statistically higher than that for RS–EPI.

## Discussion

In this study, the single-shot echo-planar imaging (SS–EPI) sequence showed better image quality of the rat sciatic nerve DTI compared with the readout-segmented echo-planar imaging (RS–EPI) sequence. Furthermore, the SS–EPI-derived DTI parameters were superior and showed stronger linear correlations with the histopathological parameters of the rat sciatic nerve compared with the RS–EPI-derived DTI parameters.

In this study, the image quality scores of the SS–EPI sequence were significantly higher than those of the RS–EPI sequence regarding the clarity of the sciatic nerve margins, sciatic nerve artifacts, and neuromuscular junction homogeneities. The differences between SS–EPI and RS–EPI results in this study can be explained by three plausible reasons. Firstly, RS–EPI is extremely sensitive to motion artifacts compared with SS–EPI. The invisible trembling after anesthesia can lead to motion artifacts, especially in small structures such as the sciatic nerve. Furthermore, the acquisition time is longer for RS–EPI compared with SS–EPI because multiple shots with shorter echo train lengths are captured along the readout direction for the *k*-space in RS–EPI (Friedli et al., 2017). This may account for the blurred margins and more obvious artifacts of the sciatic nerve in the RS–EPI images in this study. Moreover, the scanning time for SS–EPI in our study was significantly shorter compared with the scanning time for RS–EPI (4:06 min vs. 14:12 min). Shorter scanning times reduce the risk of motion artifacts. In our study, seven readout segments (14:12 min) were chosen as the upper limit to complete other functional sequences during the anesthesia time. The small size of the peripheral nerves in small experimental animals poses a challenge for MRI scanning analysis. MRI with smaller voxels enhanced the spatial resolution of the images but also introduced motion artifacts (Van der Pol et al., 2018). Furthermore, stronger signals from the smaller coils increased the motion artifacts (Gruber et al., 2018; Vergara Gomez et al., 2019).

Second, the main advantage of RS–EPI compared with SS–EPI is the significant reduction of geometric distortion caused by susceptibility artifacts (Bogner et al., 2012; Yeom et al., 2013). The performances of RS–EPI and SS–EPI are comparable when the susceptibility artifacts are less severe. Yeom et al. (2013) demonstrated that the overall image quality of RS–EPI was significantly higher than that of SS–EPI for anatomical regions prone to distortion, such as the orbit, skull base, and posterior fossa. These areas are adjacent to the paranasal sinus gas and bone tissues, which are easily prone to magnetic susceptibility artifacts. The sciatic nerve is located deep inside the muscle mass. Furthermore, differences in the magnetization rate are small between the sciatic nerve and the adjacent tissues. Therefore, magnetic sensitivity artifacts are not commonly observed in this region. Hence, SS–EPI shows better image quality. The image scores for the femur and muscles (adjacent to the air) are superior for RS-EPI compared with the SS-EPI sequence in this study for the same reasons mentioned above.

Thirdly, in this study, SS-EPI showed superior never tissue contrast. The CNR for SS-DWI was higher compared with the RS-DWI. This is because the nerve signal intensity for SS-DWI is higher compared with RS–DWI, while the signal intensities of the muscles for the two DWIs were close. Besides, the additional T2 contrast in SS–EPI DWI resulted in the T2 shine-through effect. T2 shine-through effect is advantageous in evaluating lesions with high T2 intensity that occur within the brain parenchyma or are found immediately adjacent to the brain parenchyma (Yeom et al., 2013). However, RS–EPI with shorter TE reduced the T2 contrast in the DTI images (Chen et al., 2020). In addition, the k-space coverage of SS–EPI is more efficient (Bogner et al., 2012). This might explain the better tissue contrast of rat sciatic nerve (T2 high intensity) on SS–EPI DWI compared with RS–EPI in our study. Thus, the sharper nerve edge, fewer artifacts, and better tissue contrast of SS–DWI made the ROI for sciatic nerve measurements easier to draw and more accurate. This explains why SS–EPI DTI can generate richer fibers with a more condensed bundle compared with RS–EPI DTI as well.

In our study, the ADC values for SS–EPI and RS–EPI were not significantly different and were comparable to previously published values (Bogner et al., 2012; Friedli et al., 2017). The FA and RD values for SS–EPI were significantly different from those for RS–EPI, which corroborates with the significantly higher motion artifacts and lower SNRs and T2 contrast in the rat sciatic nerve DTI with the RS–EPI sequence. These factors could affect the accuracy of the measured values and interfere with the precision of the ROI.

DTI is a useful technique for accurately assessing peripheral nerve pathology because it provides quantitative information regarding the orientation and structural features. FA quantifies the packing density of axons within a voxel and represents the degree of directed water diffusion; RD quantifies the diffusion perpendicular to the axonal orientation; AD quantifies the diffusion along the axons in parallel with the predominant fiber orientation and represents the average diffusivity in all spatial directions (Jeon et al., 2018). FA and RD values are the most stable and sensitive biomarkers among the DTI metrics to evaluate peripheral nerve regeneration (Naraghi et al., 2015; Chen et al., 2017). Previous studies on peripheral nerve repair have shown that the density and integrity of myelin increase during the process of nerve fiber regeneration, thereby resulting in higher FA and lower RD values (Chen et al., 2017; Farinas et al., 2020; Zheng et al., 2021). Conversely, nerve anisotropy (FA) values decrease, and RD values increase during peripheral nerve degeneration in healthy older individuals or individuals with peripheral nerve disease because of decreased number of myelinated fibers (Jeon et al., 2018; Lichtenstein et al., 2021). Therefore, FA and RD values are sensitive biomarkers to detect minor changes in the myelination of the peripheral nerves. In our study, the FA and RD values for both SS–EPI and RS–EPI significantly correlated with several myelin-related pathological parameters of the rat sciatic nerve. However, the overall correlation coefficients of FA and RD from the SS–EPI sequence were higher than those from the RS–EPI sequence. Furthermore, the correlation coefficients between FA and myelin thickness, FA and myelinated fiber diameter, and RD and myelin thickness for SS–EPI were statistically higher than the corresponding correlation coefficients for RS–EPI. This explains the better image quality of SS–EPI, as shown in Figure 1. These data demonstrate that SS–EPI provides better image quality of the rat sciatic nerve. Moreover, SS–EPI provided more sensitive and accurate histological information regarding the rat sciatic nerve compared with the RS–EPI.

There are several reasons for not choosing coronal planes for EPI of the rat sciatic nerve. First, a rat sciatic nerve is very small, with diameters that can range between 1 and 2.5 mm; diameters can also be 1 mm when atrophied. Accurately displaying the sciatic nerve in a single plane because of its curvature and the ROI in the coronal view can also be challenging. Partial volume effects also diminish the accuracy of the measured values. Second, the upper and lower edges of the image in the coronal view are closer to both ends of the coil. The loss of signal encountered at the coil edge can significantly affect DTI quality and evaluation. However, the cross-sectional scan is performed at the center of the coil, thereby avoiding the above-mentioned problems.

This study has a few limitations. First, since healthy adult rats were used in this study, the differences in the DTI parameters for the pathological changes in the sciatic nerve were small. In future studies, RS–EPI and SS–EPI parameters should be compared in studies examining rats with sciatic nerve injury. Second, we could not prevent the voluntary movement of live animals during MRI scanning; therefore, we performed deep anesthesia and secured the limbs with tape to prevent movement. However, this did not prevent motion artifacts in the RS–EPI sequence. In future MRI experiments, it is advised that an embedded device should be used for better immobilization of rats with reduced voluntary movements. Third, RS-EPI with various readout segments was not performed in this study. It is plausible that reducing readout segments with short acquisition times may lower the motion artifacts and improve the performance of RS–EPI in detecting rat sciatic nerve pathology. Last, the image quality scoring and ROI drawing in quantitative measurement of DTI metrics in our study were subjective to some extent. The objective assessment method should be used in the future (Jende et al., 2021, 2022).

## Conclusion

For the rat sciatic nerve DTI imaging, the SS–EPI sequence has significantly higher image quality compared with the RS–EPI sequence. The FA and RD derived from the SS–EPI sequence might be more sensitive and quantitative biomarkers to detect the histopathological change of the rat sciatic nerve.

## Data Availability Statement

The raw data supporting the conclusions of this article will be made available by the authors, without undue reservation.

## Ethics Statement

The animal study was reviewed and approved by the Institutional Animal Care and Use Committee, Jennio Biotech Co., Ltd.

## Author Contributions

YC: data collection and analysis, experiment design, and writing of the manuscript. FM and ZL: rat MRI scan and sequence optimization. YG: technical assistance. YH: sequence optimization guidance, editing, and discussion and revision of the manuscript. ZP: statistical analysis of data and creation of statistical charts. XY: pathological staining and analysis. XL and HL: full access to all the data in the study and take responsibility for the integrity of the data and the accuracy of the data analysis. All authors contributed to the article and approved the submitted version.

## Conflict of Interest

YG was employed by the company Siemens Healthineers, Guangzhou, China. The remaining authors declare that the research was conducted in the absence of any commercial or financial relationships that could be construed as a potential conflict of interest.

## Publisher’s Note

All claims expressed in this article are solely those of the authors and do not necessarily represent those of their affiliated organizations, or those of the publisher, the editors and the reviewers. Any product that may be evaluated in this article, or claim that may be made by its manufacturer, is not guaranteed or endorsed by the publisher.

## Footnotes

1
https://imagej.net
